# Nucleoside analogs treatment delay the onset of hepatocellular carcinoma in patients with HBV-related cirrhosis

**DOI:** 10.18632/oncotarget.18075

**Published:** 2017-05-22

**Authors:** Jingfeng Bi, Zheng Zhang, Enqiang Qin, Jun Hou, Shuiwen Liu, Zengmin Liu, Shuo Li, Zhenman Wei, Yanwei Zhong

**Affiliations:** ^1^ Research Center for Clinical and Translational Medicine, 302 Hospital, Beijing, 100039, China; ^2^ Infectious Disease Treatment Center, 302 Hospital, Beijing, 100039, China; ^3^ Medical Department, 302 Hospital, Beijing, 100039, China; ^4^ Medical Department, XingLong Hospital of TCM, Beijing, 100039, China; ^5^ Institute of Infectious Disease, Pediatric Liver Disease Therapy and Research Center, 302 Hospital, Beijing, 100039, China

**Keywords:** nucleoside analogs, hepatitis B, cirrhosis, hepatocellular carcinoma

## Abstract

Whether Nucleos(t)ide analogs(NA) treatment can delay the onset of HCC remains unclear. We retrospectively analyzed the clinical data of patients with HBV-related cirrhosis and HCC from 2000 to 2012. Cox proportional hazards model was used to explore the association between NA treatment and postponement of HCC development, the dependent variable was time interval from cirrhosis treatment towards the onset of HCC, and the covariates included age, sex, family history, compensation status at baseline. A total of 1155 HCC patients treated with NAs (*n =* 528, lamivudine, adefovir, entecavir) and non NA (*n =* 627) for more than 24 months before the occurrence of HCC were incorporated into the cohort. Compared with the non-NA group, NAs therapy was associated with delaying the onset of HCC in patients with cirrhosis. Significant factors were: adefovir treatment (*n =* 181; *p* = 0.0072; HR: 0.792; 90% CI: 0.687–0.914), entecavir treatment (*n =* 83; *p* = 0.0068; HR: 0.716; 90% CI: 0.585-0.877), lamivudine switched to adefovir treatment (*n =* 95, *p* = 0.0808; HR: 0.822; 90% CI: 0.684 to 0.989). But Lamivudine monotherapy was not a significant factor (*n =* 102; *p* = 0.6877; HR: 1.045; 90% CI: 0.873–1.250). Long-term NA treatment (> 6 months, except for lamivudine monotherapy) can delay the onset of HCC in patients with HBV-related cirrhosis, and applying high barrier NA to resistance is important in these patients.

## INTRODUCTION

Hepatitis B virus (HBV) infection is a major public health issue worldwide, about 2 billion people were infected with HBV globally, and approximately 240 million people are chronically infected with HBV [1]. The annual incidence of hepatocellular carcinoma (HCC) in HBV carriers was 0.5%, and the annual risk of HCC in patients with cirrhosis has been reported 2.5% to 6% [2–5]. For cirrhosis and HCC patients, 30% to 45% of them were caused by HBV infection globally [6, 7], but in the areas with high HBV prevalence (Asia and Africa), 60% to 80% of patients with HCC has an etiology of HBV infection [8, 9]. Worldwide, about 650000 people die from HBV-related liver failure, cirrhosis and HCC each year [6], making HBV infection a great economic and health burden. Antiviral therapy has been a rational approach to reduce the development of HCC, as the risk for HCC is higher for patients with persistent high viral replication [10].

The data of the antiviral therapy showed that both IFN-α and NA treatment may decrease HCC incidence, especially for those sustained responders [11, 12]. NA inhibits the HBV replication by competing for incorporation into viral DNA. Benefits of NA treatment include long-term HBV DNA suppression and reduction in hepatic fibrosis, hepatic decompensation, and liver-related mortality. Some studies reported that NA improved survival and decreased the risk of HCC recurrence after liver resection [13, 14]. However, whether NA treatment can delay the progression of HCC among patients with HBV-related cirrhosis remains unclear. This study, we applied a historical cohort study to evaluate the effect of NA treatment on the interval time from cirrhosis to HCC onset in patients who developed HCC with HBV-related cirrhosis etiology.

## RESULTS

### Patient characteristics

From January 2000 to March 2012, a total of 15,540 patients’ medical records diagnosed with HCC in Beijing 302 Hospital were studied. Among them, 11743 cases were HBV-related hepatocellular carcinoma, the rest of them were hepatitis C, Hepatitis B and C co-infection, or non-hepatitis virus-related hepatocellular carcinoma.

**Figure 1:**
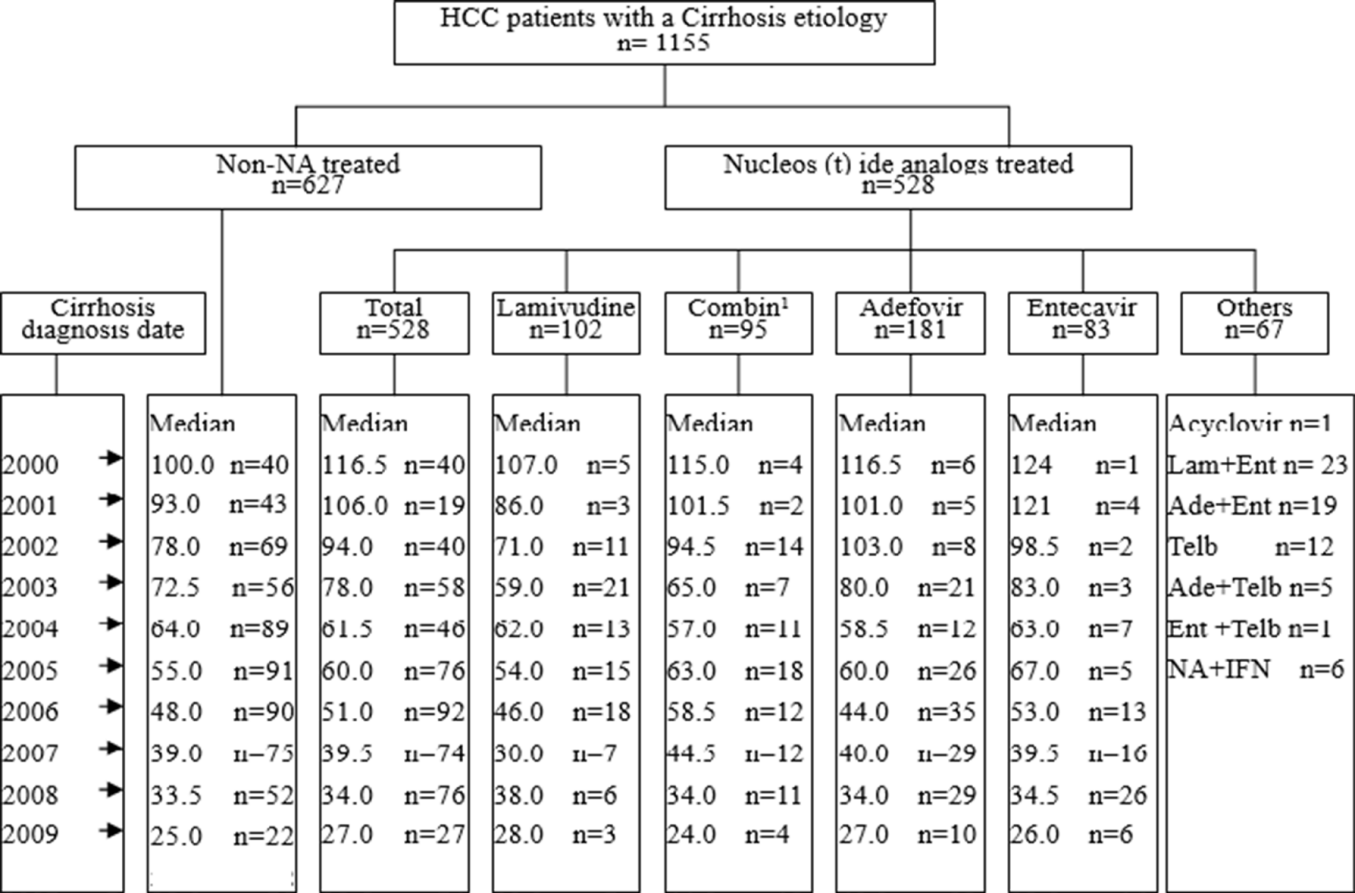
Patient characteristics Combin: lamivudine sequential adefovir. Median: the median of interval months from cirrhosis to the diagnosis of HCC n: case number.

Of the 11743 patients with confirmed HCC, 1155 cases were in line with the requirements of the present study (Figure 1). The others were excluded from the study, the reason as follows: some cases had not pathological diagnosis results; some patients were treated by IFN monotherapy; some patients developed to hepatocellular carcinoma without cirrhosis stage; the interval time in some patients between the initiation date of NA therapy and the diagnosis date of HCC was less than 24 months.

Among the 1155 patients, non-NA treated group was 627 and NA treatment group was 528, including lamivudine (LAM) treated 102, lamivudine sequential adefovir (ADV) 95, adefovir 181, entecavir (ETV) 83, the rest of them 67 (Figure 1)

As shown in Table 1, among the 102 patients treated with LAM, 14 of them were male and 88 were female. The mean age (± standard deviation[SD]) was 48.12 ± 9.10 years and the median age was 47.5 (28–67) years; Of the181 patients treated with ADV, 40 of them were male and 141 were female, their mean age was 49.04 ± 8.65 years and the median age was 50 (26–72) years. In the 83 treated with ETV, 8 of them were male and 75 were female.The mean age was 47.07 ± 8.25 years and the median age was 47 (30–69) years; Of the 95 patients treated with LAM+ADV, 18 were male and 77 were female, whose mean age was 46.79 ± 9.58 years and the median age was 46 (26–68) years. The mean of time interval between cirrhosis treatment and onset of HCC in the non NA treated group was 59.02 ± 29.66months; in NA treated group was 57.22 ± 26.04months. Of the LAM, ADV, ETV, LAM sequential ADV and other NA treatment groups, the mean of time interval were 58.12 ± 23.98, 55.58 ± 25.56, 50.70 ± 24.84, 60.88 ± 25.88, 63.15 ± 30.24 months, respectively. Because the data of HBV DNA or ALT level was incomplete in some patients, these data was not presented in Table 1.

**Table 1:** Patient demographics

Factor		NA-treated						Non-treated *N =* 627	*p*^1^
		LAM *N =* 102	ADV *N =* 181	ETV *N =* 83	LAM+ ADV *N =* 95	Others *N =* 67	Total *N =* 528		
**Sex**	Female/Male	88/14	141/40	75/8	77/18	52/15	433/95	527/100	0.3557
**Age**	Mean ± Sd	48.12 ± 9.10	49.04 ± 8.65	47.07 ± 8.25	46.79 ± 9.58	45.06 ± 10.18	47.64 ± 9.12	49.19 ± 10.00	0.0062
	Median (Min-Max)	47.5 (28–67)	50 (26–72)	47 (30–69)	46 (26–68)	47 (15–61)	48 (15–72)	49 (15–78)	
age < 40		18	31	16	21	15	101	102	0.0682
40 ≤ age < 60		72	129	62	64	49	376	439	
age ≥ 60		12	21	5	10	3	51	86	
**Family history**									0.0009
HCC		55	77	34	48	31	245	351	
Cirrhosis		30	66	36	32	22	186	155	
HBV		7	15	5	7	9	43	60	
No history		10	23	8	8	5	54	61	
**Compensation status**		30/72	54/127	22/61	33/62	28/39	167/361	178/449	0.2308
**Time**^2^ **(Month)**	Mean ± Sd	58.12 ± 23.98	55.58 ± 25.56	50.70 ± 24.84	60.88 ± 25.88	63.15 ± 30.24	57.22 ± 26.04	59.02 ± 29.66	0.2381
	Median (Min-Max)	54 (24–139)	53 (24–132)	42 (24–125)	57 (24–135)	55 (24–140)	53 (24–140)	55 (24–140)	

1.p, Non-treated vs Total NA treated.

2.Time, the interval time between cirrhosis treatment and HCC diagnosis date.

### Log-rank test of different NA treated groups vs non-NA treated group

**Figure 2:**
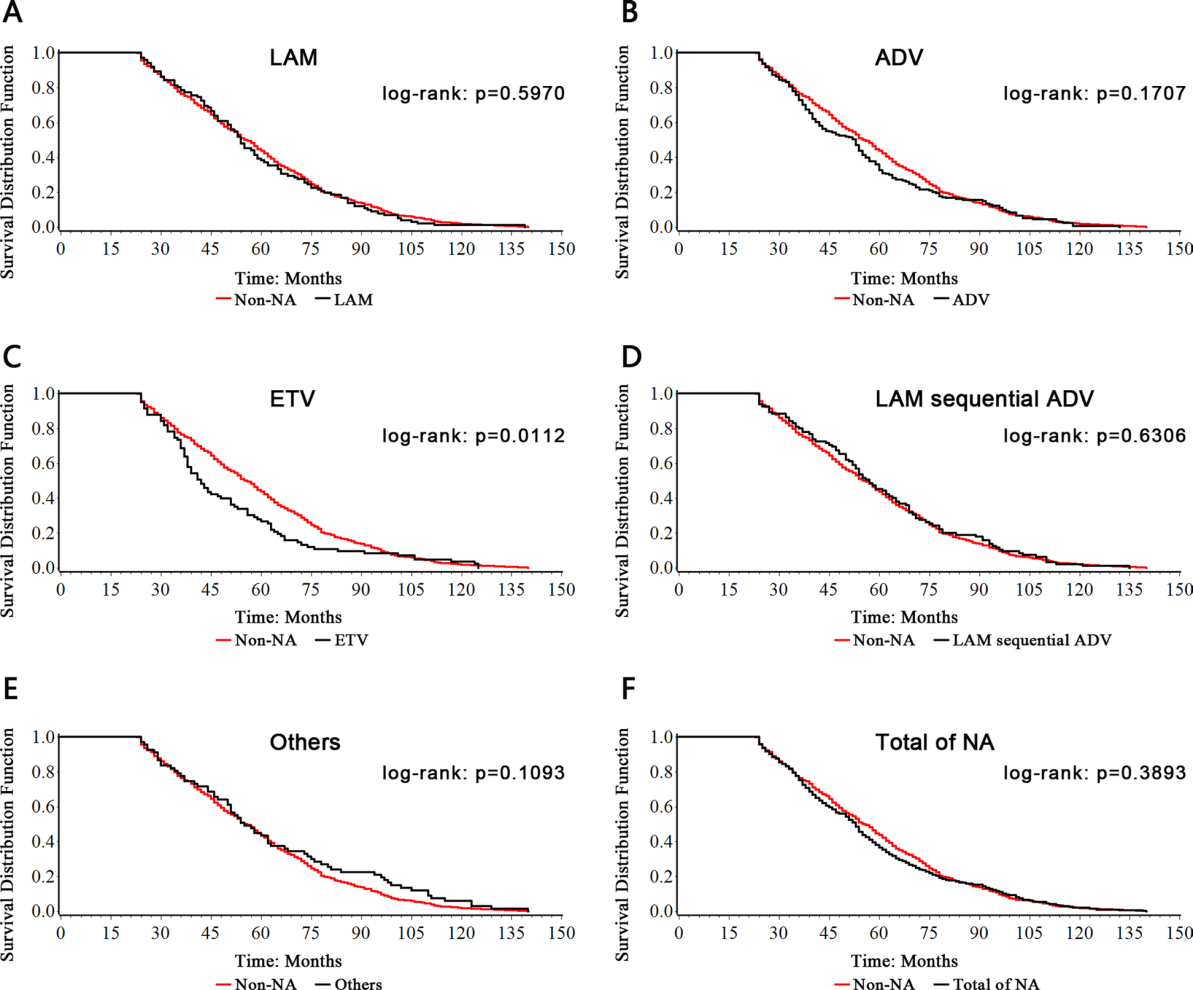
Long-rank test of different NA treated groups vs Non-NA group

The Log-rank test results of different NA treated groups versus non-NA treated group showed that the survival curves had multiple intersections (Figure 2), which did not meet the proportional hazards(the prerequisite for COX regression analysis).

In this study, the cases distributed from 2000 to 2009 for their treatment date of cirrhosis, and observed till March 2012, that means the patients included in 2000 were involved in the analysis for up to 13 years, while those cases in 2009 involved were only observed less than 3 years, which indicated different treatment time of cirrhosis had different proportional hazards. Based on this reason, we conducted the year of treatment as a stratification variable into the COX regression model.

### The effect of NAs treatment on the interval time from cirrhosis to HCC

In the COX regression model, sex, age, family history, compensation status were taken as covariates, the treatment date of cirrhosis was introduced as a stratification factor to explore the effect of different NA drugs on the occurrence time of HCC. The results indicated that sex (male as the reference, *n =* 195; female: *n =* 950, *p =* 0.1816, HR: 1.115, 90% CI: 0.975 to 1.276), age (age < 40 as the reference, *n =* 203; 40 ≤ age < 60: *n =* 815, *p =* 0.6087, HR: 1.043, 90% CI: 0.912 to 1.193; age ≥ 60: *n =* 137, *p =* 0.5861, HR: 1.066, 90% CI: 0.879 to 1.294), family history, (non history as the reference, *n =* 115; HCC: *n =* 596, *p =* 0.2130, HR: 0.915, 90% CI: 0.814 to 1.029; cirrhosis: *n =* 341, *p =* 0.7908, HR: 0.971, 90% CI: 0.812 to 1.162; HBV: *n =* 103, *p =* 0.6441, HR: 0.952, 90% CI: 0.801 to 1.133); compensation status (decompensation status as the reference, *n =* 345; compensation status: *n =* 810, *p =* 0.1058, HR: 1.114, 90% CI: 0.998 to 1.244) were not significant factors in the Cox model. As presented in Table 2, NAs therapy (*n =* 528; *p =* 0.0004; HR: 0.810; 90% CI: 0.721–0.911) could notably prolong the interval time from cirrhosis to HCC compared with non-NA treatment group (*n =* 627; HR=1), however lamivudine monotherapy (*n =* 102; *p =* 0.6877; HR: 1.045; 90% CI: 0.873–1.250) is not a significant factor. Significantly, the interval time from cirrhosis therapy to HCC onset was longer in adefovir, entecavir and LAM+ADV treated patients compared with non-NA treated patients, in other words, adefovir monotherapy(*n =* 181; *p =* 0.0072; HR: 0.792; 90% CI: 0.687–0.914),entecavir monotherapy (*n =* 83; *p =* 0.0068; HR: 0.716; 90% CI: 0.585–0.877) and LAM+ADV((lamivudine > 6 months and switching to adefovir > 6 months, *n =* 95, *p* = 0.0808; HR: 0.822; 90% CI: 0.684 to 0.989) could be taken as significant factors in this model. The hazard ratio of HCC onset in other NA treated groups(67 patients) was also significant lower than non-NA treated groups (*n =* 67, *p =* 0.0166; HR: 0.731; 90% CI: 0.589 to 0.906), and those NA treated groups included 23 patients who sequentially received lamivudine and entecavir, 19 patients who sequentially received adefovir and entecavir, 5 patients who sequentially received adefovir and telbivudine, one patient who sequentially received entecavir and telbivudine, 12 patients with monotherapy of telbivudine, 6 patients who sequentially received nucleoside and interferon.

**Table 2:** The results of COX regression analysis

Factor	Group	N	HR	90% CI		*P* value
**Sex**	Male	195	1.000 (Reference)			
	Female	960	1.115	0.975	1.276	0.1816
**Age**	age < 40	203	1.000 (Reference)			
	40 ≤ age < 60	815	1.043	0.912	1.193	0.6087
	age ≥ 60	137	1.066	0.879	1.294	0.5861
**family history**	Non history	115	1.000 (Reference)			
	HCC	596	0.915	0.814	1.029	0.2130
	Cirrhosis	341	0.971	0.812	1.162	0.7908
	HBV	103	0.952	0.801	1.133	0.6441
**Compensation status**	Decompensation	345	1.000 (Reference)			
	Compensation	810	1.114	0.998	1.244	0.1058
**time lengths from cirrhosis treatment to HCC onset**	Non-treated	627	1.000 (Reference)			
	Total	528	0.810	0.721	0.911	0.0004
	LAM	102	1.045	0.873	1.250	0.6877
	ADV	181	0.792	0.687	0.914	0.0072
	ETV	83	0.716	0.585	0.877	0.0068
	LAM+ADV	95	0.822	0.684	0.989	0.0808
	Others	67	0.731	0.589	0.906	0.0166

## DISCUSSION

Previous studies showed that NA treatment could decrease the incidence of HCC [15–19]. However, whether NA treatment can delay the onset of HCC remains unclear. To our knowledge, this is the first study to explore whether NA long-term treatment could slow down the progression of cirrhosis towards HCC. Since there were already sufficient evidence to support antiviral therapy benefits patients with HBV-related cirrhosis, implementing a prospective placebo-controlled or blank controlled clinical study would face huge ethical issues, a retrospective study was conducted here. Using the Cox proportional hazards model, we found the hazards ratios in patients treated with adefovir or entecavir monotherapy, sequentially received lamivudine and adefovir were significant lower as compared to non-NA treated groups. But the hazards ratio difference was not observed in the patients with lamivudine monotherapy.

“AASLD practice guidelines- Chronic Hepatitis B: Update 2009” quoted Liao’s double blind, randomized, placebo-controlled study, which demonstrated that lamivudine reduced the incidence of HCC in chronic hepatitis B (CHB) (lamivudine: 17:436, 3.9%; placebo: 16:215,7.4%; *p =* 0.047) [20]. In order to understand the impact of HBV polymerase mutation on antiviral drug resistance, we investigated the virologic and clinical features of HBV mutations in those patients suffered NAs treatment. We found that the patients suffered LAM monotherapy had a high ratio of resistance mutation than other patients, which might be the reason of lamivudine monotherapy can’t delay the onset of HCC in this study. We suggested that high genetic barrier NA should be used first in the patients with HBV-related cirrhosis.

Previous studies had demonstrated that male gender, family history of HCC, older age, history of reversions from anti-HBe to HBeAg, alcoholics, diabetes, aflatoxin, Genotype B or C, persistent liver inflammation and co-infection with HCV were risk factors for HCC [21–28]. A few recent follow-ups on large cohorts of prospective carriers from Asia identified the presence of HBeAg and high levels of HBV DNA and ALT as independent risk factors for the subsequent development of cirrhosis and HCC [29–30].Although there was no evidence indicated that these factors had an impact on interval time between cirrhosis and HCC in patients with HBV-related cirrhosis etiology, but the analysis of these factors will make the results more reliable. In this study, we investigated the influence of age, sex and family history on interval time between cirrhosis and HCC, but there were no significant statistical differences in the three factors. As presented in Table 2, the hazard ratio for the three factors was closed to 1, it seems none of them has significant effect on interval time between cirrhosis and HCC.

The data of HBeAg seroconversion, alcoholics, diabetes, aflatoxin, Genotype, the degree of the liver tissues inflammation, HBV DNA, ALT in this retrospective study were not presented, the reason was most of these information in medical records was imperfect in the duration of 13 years.

Of the above-mentioned factors, we focused on the variables which would affect the doctor’s treatment strategy (anti-virus treatment or no anti-virus treatment), and we considered those variables which had no effect on the doctor’s treatment strategy could keep balance between the two groups in those relatively large samples.

Based on the “EASL 2017 Clinical Practice Guidelines on the management of hepatitis B virus infection” [31], the typical indication for treatment requires high HBV DNA level, elevated ALT and/or at least moderate histological lesions, while all cirrhotic patients with detectable HBV DNA should be treated regardless of ALT levels. The results of the COX regression analysis indicated that the hazards ratios in patients treated with NA, especially with monotherapy of adefovir or entecavir, sequentially received lamivudine and adefovir were significant lower than non-NA treated group. The long-term administration of NA with high barrier to resistance represents the treatment of choice in the patients with compensated or decompensated cirrhosis. NA with high barrier to resistance can halt progression of liver disease, and can also result in a significant improvement of histological necroinflammation and fibrosis [31].

## MATERIALS AND METHODS

### Study populations

The clinical data of patients with HCC were collected from the Beijing 302 hospital medical records system database from 2000 to 2012. Inclusion criteria: NA treated (treatment > 6 months) or non-NA treated patients with a HCC diagnosis before March 2012 and a confirmed HBV-related cirrhosis treatment for the period between January 2000 (as lamivudine was approved in China in June 1999) and December 2009 (the time from cirrhosis to HCC was at least two years). A medical examination was performed with imaging, histopathology or serological markers. The interval between nucleos(t)ide analogs initiation date and HCC diagnosis date was at least 24 months. Exclusion criteria: patients with co-infection with hepatitis C, D, E virus or human immunodeficiency virus; patients with alcoholic or autoimmune cirrhosis, drug induced liver injury(DILI), parasite, inherited liver disease, metabolic liver disease, nonalcoholic fatty liver disease (NAFLD) and patients continued alcohol consumption after treatment of cirrhosis.

### Study design and survey content

Survey data included patients’ age at baseline (the date of cirrhosis treatment), sex, family history (HCC, cirrhosis, HBV, or no history), date of cirrhosis treatment, date of HCC diagnosis, treatment (lamivudine, entecavir , and adefovir treated or non-NA treated); HBV DNA and ALT level at baseline; compensation status at baseline (Decompensation: gastrointestinal bleeding, hepatic encephalopathy, ascites).

### Statistical analysis

Multivariable Cox proportional hazards regression models to assess the relation between NAs treatment and the delaying onset of HCC were constructed. Cox regression analysis was used to identify independent risk factors for delaying onset of HCC. The models included the base-line covariates age (continuous), sex (categorical; female, male), treatment (categorical; dummy variable; non-NA treated, lamivudine, entecavir, adefovir, independent, joint or sequential therapy), family history (categorical; dummy variable; family history of HCC, cirrhosis, HBV, or no history), date of cirrhosis treatment (stratification; accurate to year), compensation status (categorical; compensation, decompensation). The statistic analyses were conducted using SAS 9.2 (SAS Institute Inc), all statistical tests were 2-sided with *p* < 0.1 deemed as statistical significant, and the odds ratios (ORs) and 90% confidence intervals (CIs) were calculated to assess the relative risk confidence.

### Ethics statement

The study protocol was approved by the Beijing 302 Hospital Research Ethnics Committee, and written informed consents for therapy and study were obtained from each patient.
